# Crystal structure and Hirshfeld surface analysis of two (*E*)-*N*′-(*para*-substituted benzyl­idene) 4-chloro­benzene­sulfono­hydrazides

**DOI:** 10.1107/S205698901801592X

**Published:** 2018-11-16

**Authors:** Akshatha R. Salian, Sabine Foro, B. Thimme Gowda

**Affiliations:** a Department of Chemistry, Mangalore University, Mangalagangotri-574 199, India; b Institute of Materials Science, Darmstadt University of Technology, Alarich-Weiss-Str. 2, D-64287, Darmstadt, Germany; c Karnataka State Rural Development and Panchayat Raj University, Gadag-582101, India

**Keywords:** crystal structure, hydrazones, *N′*-(aryl­idene)aryl­sulfono­hydrazides, hydrogen bonding, graph-set motif, Hirshfeld surface analysis, fingerprint plots

## Abstract

Two (*E*)-*N*′-(*p*-substituted benzyl­idene)-4-chloro­benzene­sulfono­hydrazides, namely, (*E*)-4-chloro-*N*′-(4-chloro­benzyl­idene)benzene­sulfono­hydrazide, C_13_H_10_Cl_2_N_2_O_2_S, (I), and (*E*)-4-chloro-*N*′-(4-nitro­benzyl­idene)benzene­sulfono­hydrazide, C_13_H_10_ClN_3_O_4_S, (II), have been synthesized, characterized and their crystal structures studied to explore the effect of the nature of substituents on the structural parameters. Compound (II) crystallized with two independent mol­ecules [(II*A*) and II*B*)] in the asymmetric unit. In both compounds, the configuration around the C=N bond is *E*. The mol­ecules are twisted at the S atom with C—S—N—N torsion angles of −62.4 (2)° in (I), and −46.8 (2)° and 56.8 (2)° in the mol­ecules *A* and *B* of (II). The 4-chloro­phenyl­sulfonyl and 4-substituted benzyl­idene rings form dihedral angles of 81.0 (1)° in (I), 75.9 (1)° in (II*A*) and 73.4 (1)° in (II*B*). In the crystal of (I), mol­ecules are linked *via* pairs of N—H⋯O hydrogen bonds, forming inversion dimers with an *R*_2_^2^(8) ring motif. The dimers are linked by C—Cl⋯π inter­actions, forming a three-dimensional structure. In the crystal of (II), mol­ecules are linked by C—H⋯π inter­actions and N—H⋯O hydrogen bonds, forming –*A*–*B*–*A*–*B*– chains along the *c*-axis direction. The chains are linked *via* C—H⋯O and C—H⋯π inter­actions, forming layers parallel to the *bc* plane. Two-dimensional fingerprint plots show that the most significant contacts contributing to the Hirshfeld surface for (I) are H⋯H contacts (26.6%), followed by Cl⋯H/H⋯Cl (21.3%), O⋯H/H⋯O (15.5%) and Cl⋯C/C⋯Cl (10.7%), while for (II) the O⋯H/H⋯O contacts are dominant, with a contribution of 34.8%, followed by H⋯H (15.2%), C⋯H/H⋯C (14.0%) and Cl⋯H/H⋯Cl (10.0%) contacts.

## Abstract 2

The crystal structures of (*E*)-4-chloro-*N*′-(4-chloro­benzyl­idene)benzene­sulfono­hydrazide and (*E*)-4-chloro-*N*′-(4-nitro­benzyl­idene)benzene­sulfono­hydrazide have been studied to investigate the effect of substituents on the structural parameters. The two-dimensional fingerprint plots of these two *p*-substituted compounds indicate that in the 4-chloro-substituted compound, the largest contribution to the Hirshfeld surface comes from the H⋯H contacts (26.6%), in contrast to the 34.8% contribution of the O⋯H/H⋯O contacts in the 4-nitro-substituted compound.

## Chemical context   

In the field of synthetic chemistry, hydrazones are frequently used as nucleophiles and electrophiles (Ogawa *et al.*, 2004 ▸). They also play an important role in organic synthesis as one of the reaction inter­mediates due to their ring-closure reactions (Rollas & Küçükgüzel, 2007 ▸). Hydrazones have drawn considerable attention in the field of coordination chemistry (Weber *et al.*, 2007 ▸). They also find various industrial applications (Reis *et al.*, 2013 ▸) and exhibit a wide spectrum of biological activities (da Silva *et al.*, 2011 ▸). Aryl­sulfonyl-hydrazones have shown anti­tumour activity in addition to their role as a versatile source of diazo compounds in many metal-catalysed and metal-free reactions (Hashemi, 2012 ▸). In a continuation of our efforts to explore the effect of site and nature of substituents on the crystal structures of 4-chloro-aryl­sulfono­hydrazide derivatives (Salian *et al.*, 2018 ▸), we report herein the synthesis, characterization, crystal structures and Hirshfeld surface analysis of the title compounds, (I) and (II), and compare them with those of the recently reported structures of (*E*)-4-chloro-*N*′-(benzyl­idene) benzene­sulfono­hydrazide (III), (*E*)-4-chloro-*N*′-(2-methyl­benzyl­idene)benzene­sulfono­hydrazide (IV) and (*E*)-4-chloro-*N*′-(4-methyl­benzyl­idene)benzene­sulfono­hydrazide (V) (Salian *et al.*, 2018 ▸).
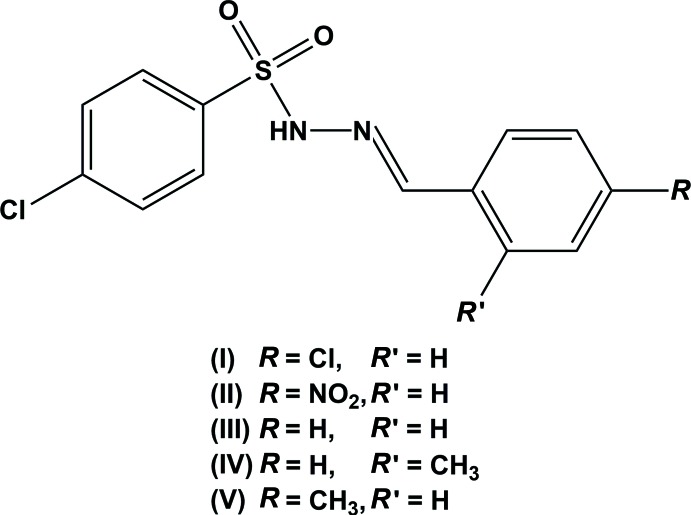


## Structural commentary   

**Figure 1:**
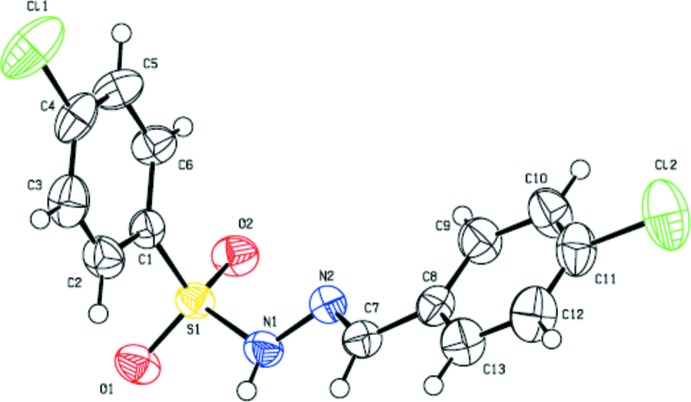
Mol­ecular structure of (I), with the atom labelling and displacement ellipsoids drawn at the 50% probability level.

**Figure 2:**
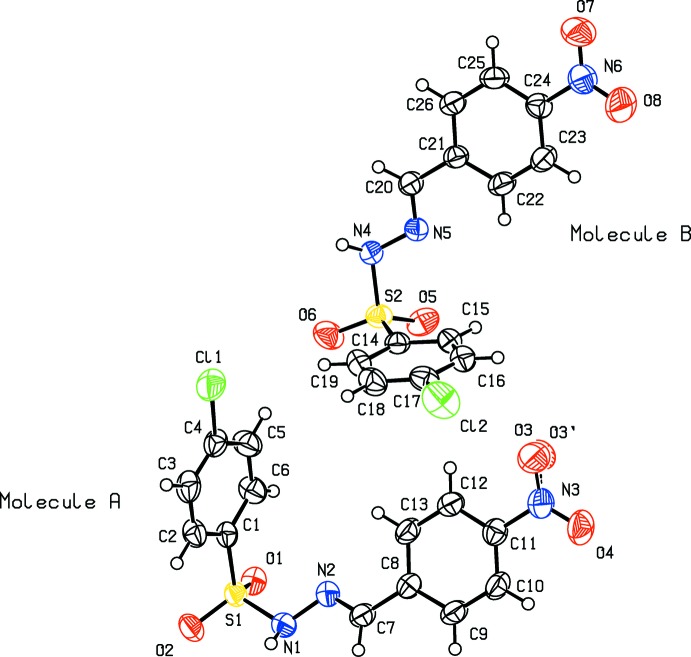
Mol­ecular structure of (II), with the atom labelling and displacement ellipsoids drawn at the 50% probability level.

Compound (I), crystallizes in the triclinic crystal system, space group *P* 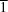, with one mol­ecule in the asymmetric unit (Fig. 1 ▸), while compound (II) crystallizes in the monoclinic crystal system, space group *P*2_1_/*c*, with two independent mol­ecules [(II*A*) and (II*B*)] in the asymmetric unit (Fig. 2 ▸). For both the compounds, the configuration about the C=N bond is *E* and the conformations of the N—H and C—H bonds in the hydrazone segments are *syn* to each other.

The C=N bond lengths of 1.269 (3), 1.269 (3) and 1.269 (3) Å in (I), (II*A*) and (II*B*), and the N—N bond lengths of 1.388 (2) 1.397 (3) and 1.390 (2) Å in (I), (II*A*) and (II*B*), respectively, indicate the delocalization of the *π*-electron density over the hydrazone part of the mol­ecules. The other bond lengths are in close agreement with those of the parent compound (III), and the *ortho*-methyl (IV) and *para*-methyl (V) derivatives (Salian *et al.*, 2018 ▸). Selected geometrical parameters of compounds (I)–(V) are compared in Table 1 ▸ (Salian *et al.*, 2018 ▸).

In the title compounds the mol­ecules are twisted at the S atom with C—S—N—N torsion angles of −62.4 (2)° in (I), and −46.8 (2) and 56.8 (2)° in (II*A*) and (II*B*), respectively. The respective S—N—N=C torsion angles of 158.9 (2)° in (I), and 171.4 (2) and −165.3 (2)° in (II*A*) and (II*B*), denote the non-planarity of the sulfono­hydrazide parts of the mol­ecules. However, the N—N—C—C torsion angles of 175.0 (2)° in (I), and −175.9 (2) and 178.2 (2)° in (II*A*) and (II*B*), indicate near coplanarity of the hydrazide units with the benzyl­idene rings. The dihedral angles between the 4-chloro-substituted phenyl­sulfonyl ring and 4-substituted benzyl­idene ring are 81.0 (1)° in (I), and 75.9 (1) and 73.4 (1°) in mol­ecules *A* and *B* of compound (II). In comparison, the corresponding values in compounds (III), (IV) and (V) are 78.4 (2), 74.8 (2) and 76.9 (1)°, respectively (see Table 1 ▸). In (II) the *A* and *B* mol­ecules are linked by a C—H⋯Cl inter­action (Table 3 ▸).

## Supra­molecular features   

**Figure 3:**
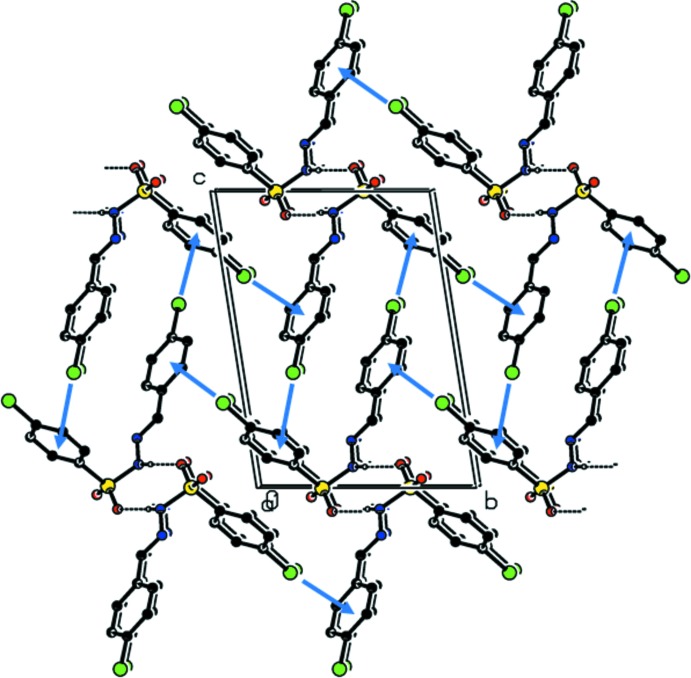
Crystal packing of (I), viewed along the *a* axis, with hydrogen bonds (Table 2 ▸) shown as dashed lines and C—Cl⋯π inter­actions as blue arrows. C-bound H atoms have been omitted.

The pattern of the hydrogen-bonding inter­actions in the crystal structures of (I) and (II) are different. In the crystal of (I), mol­ecules are linked by pairs of N—H⋯O hydrogen bonds, forming inversion dimers enclosing 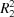
(8) loops (Fig. 3 ▸, Table 2 ▸). The dimers are linked by C—Cl⋯π inter­actions, forming a three-dimensional arrangement (Fig. 3 ▸). This is very similar to the situation observed in the crystal of compound (V) [(*E*)- 4-chloro-*N*′-(4-methyl­benzyl­idene)benzene­sulfono­hydrazide; Salian *et al.*, 2018 ▸].

**Figure 4:**
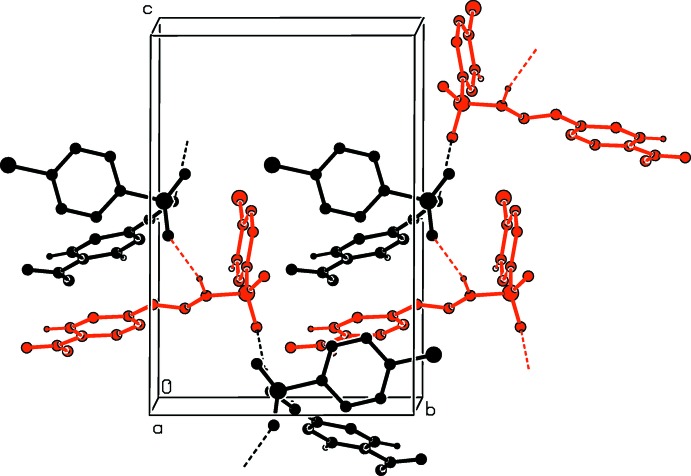
A partial view along the *a* axis of the crystal packing of (II), with hydrogen bonds (Table 3 ▸) shown as dashed lines. H atoms not involved in these inter­actions have been omitted. Colour code: black *A* mol­ecules; red *B* mol­ecules.

**Figure 5:**
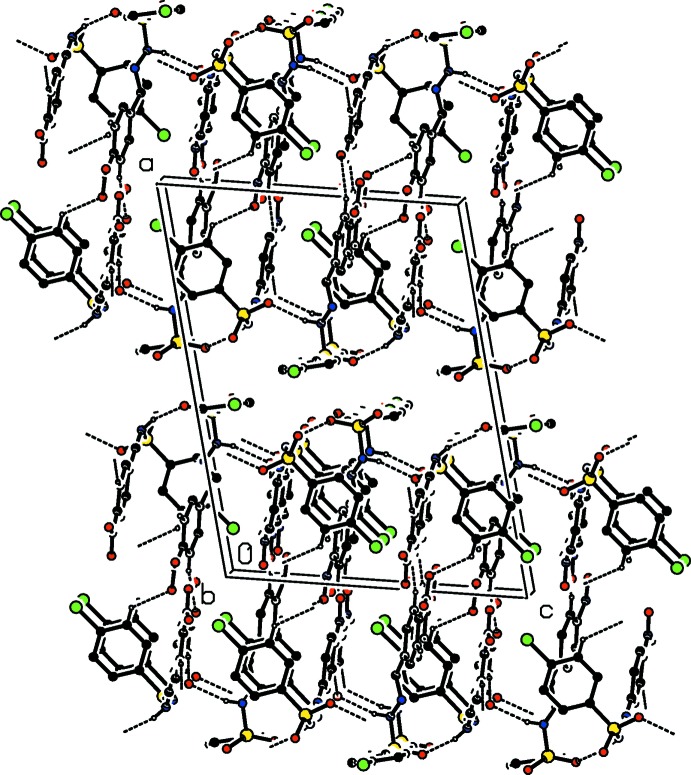
Crystal packing of (II), viewed along the *b* axis, with hydrogen bonds shown as dashed lines. H atoms not involved in these inter­actions have been omitted.

Replacement of the 4-chloro group in (I) by the 4-nitro group to produce compound (II) introduces C—H⋯O inter­actions, which stabilize the crystal packing (Table 3 ▸ and Figs. 4 ▸ and 5 ▸). The N—H⋯O hydrogen bond involving the sulfonyl O atom and the amino H atom of the hydrazide segment between the *A* and *B* mol­ecules results in the formation of –*A*–*B*–*A*–*B*– chains propagating along the *c*-axis direction (Fig. 4 ▸). The chains are linked by C—H⋯O inter­actions involving O atoms O4 in (II*A*) and O5 in (II*B*) and O7 of the nitro group and the aromatic hydrogen atoms *ortho* to the Cl or NO_2_ group. The sulfonyl O atom of (II*B*), *i.e.* O5, shows bifurcated hydrogen bonding, one with the amino H atom of the hydrazide segment and the other with one of the aromatic H atoms (H25), adjacent to the nitro group. These inter­actions link the chains, forming layers lying parallel to the *bc* plane (Table 3 ▸ and Fig. 5 ▸).

## Hirshfeld surface analysis   

**Figure 6:**
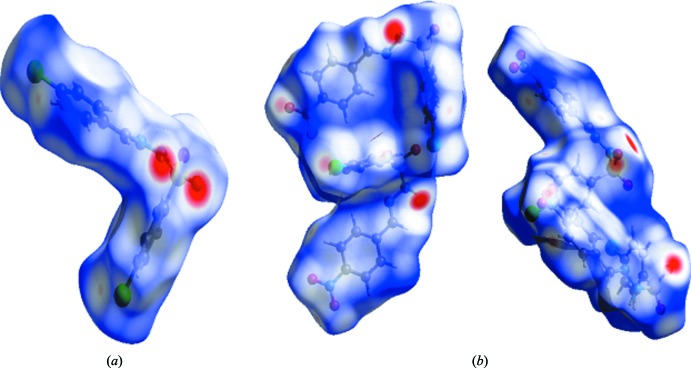
(*a*) View of the Hirshfeld surface mapped over *d*_norm_ for (I); (*b*) two views of the Hirshfeld surface mapped over *d*_norm_ for (II).

Hirshfeld surfaces and two-dimensional fingerprint plots were generated for the two substituted compounds (I) and (II) using *CrystalExplorer* (Turner *et al.*, 2017 ▸) to visualize the inter­molecular inter­actions, to investigate the impact of each kind of inter­molecular contact on the crystal packing and to study the relative strengths of the different inter­actions in the two compounds. The mol­ecular Hirshfeld surfaces were generated using a standard (high) surface resolution. *d*_i_ and *d*_e_ are the contact distances from the Hirshfeld surface to the nearest atom inside and outside, respectively [Fig. 6 ▸(*a*) for (I) and Fig. 6 ▸(*b*) for (II)]. The strong hydrogen bonds appear as dark-red spots and weak inter­actions as light-red spots on the *d*_norm_ surface (McKinnon *et al.*, 2004 ▸; Spackman & Jayatilaka, 2009 ▸).

**Figure 7:**
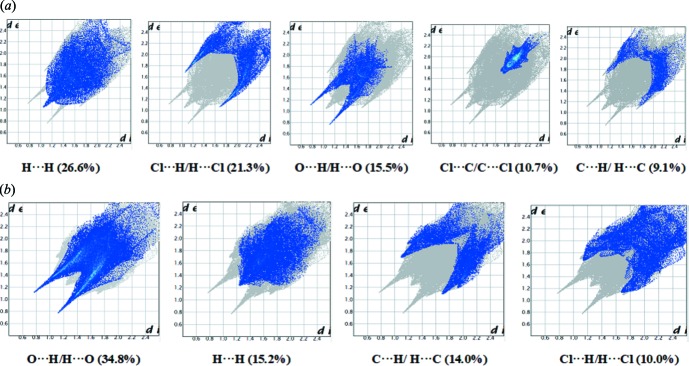
Two-dimensional fingerprint plots for (*a*) (I) and (*b*) (II). *d*_i_ is the closest inter­nal distance from a given point on the Hirshfeld surface and *d*_e_ is the closest external contact.

**Figure 8:**
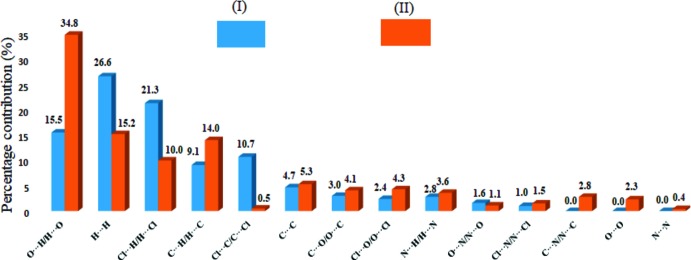
Qu­anti­tative results of different inter­molecular inter­actions contributing to the Hirshfield surfaces of (I) and (II).

Comparison of fingerprint plots for various atom–atom inter­actions show that the percentage contributions of these inter­actions to the Hirshfeld surfaces vary significantly from (I) to (II). The major contribution to the Hirshfeld surface in (I) is from H⋯H contacts (26.6%), followed by Cl⋯H/H⋯Cl (21.3%), O⋯H/H⋯O (15.5%), Cl⋯C/C⋯Cl (10.7%) and C⋯H/ H⋯C (9.1%) [Fig. 7 ▸(*a*)], while in (II), as a result of C—H⋯O inter­actions, O⋯H/H⋯O contacts are dominant and serve as the major contributors (34.8%) in the crystal packing, followed by H⋯H contacts (15.2%), C⋯H/ H⋯C (14.0%) and Cl⋯H/H⋯Cl (10.0%) [Fig. 7 ▸(*b*)]. The Cl⋯C/C⋯Cl contribution to the *d*_norm_ surface is almost negligible (0.5%) in (II). However, C⋯C, H⋯N/N⋯H and C⋯O/O⋯C contacts make very similar contributions in the two compounds, their respective contributions being 4.7, 2.8, 3.0%, in (I) and 5.3, 3.6 and 4.1% in (II). Two pairs of symmetrical, long narrow spikes are present at *d*_i_ + *d*_e_ ∼2.2 Å for the O⋯H/H⋯O contacts in the fingerprint plots of (I) and (II) and these values are very close to the H⋯*A* distances for the N—H⋯O hydrogen bonds observed in the crystal structures (Tables 2 ▸ and 3 ▸). The contributions of the other weak inter­molecular contacts to the Hirshfeld surfaces are: Cl⋯N/N⋯Cl (1.0 and 1.5%), C⋯N/N⋯C (0.0 and 2.8%), O⋯O (0, 2.3%), N⋯N (0, 0.4%) in (I) and (II), respectively. The result of the qu­anti­tative analysis of all types of inter­molecular contacts present in (I) and (II) is summarized in Fig. 8 ▸.

## Database survey   

The structures reported in the literature similar to the title compounds include (*E*)-*N*′-(4-chloro­benzyl­idene)-*p*-toluene­sulfono­hydrazide 0.15-hydrate (Kia *et al.*, 2009*a* ▸), (*E*)-*N*′-(4-chloro­benzyl­idene)-*p*-toluene­sulfono­hydrazide (Balaji *et al.*, 2014 ▸), (*E*)-*N*′-(4-bromo­benzyl­idene)-*p*-toluene­sulfono­hydra­zide (Kia *et al.*, 2009*b* ▸], (*E*)-*N*′-(4-nitro­benzyl­idene)benzene­sulfono­hydrazide (Hussain *et al.*, 2017*a* ▸) and (*E*)-4-methyl-*N′*-(4-nitro­benzyl­idene)benzene­sulfono­hydrazide (Hussain *et al.*, 2017*b* ▸). In all of these structures, inter­molecular N—H⋯O hydrogen bonds link neighbouring mol­ecules to form chains, which are linked by C—H⋯O hydrogen bonds. There are also inter­molecular *π*–*π* inter­actions present, which further stabil­ize the crystal structures.

## Synthesis and crystallization   

**Synthesis of 4-chloro­benzene­sulfono­hydrazide**

4-Chloro­benzene­sulfono­hydrazide was synthesized by a recently reported procedure (Salian *et al.*, 2018 ▸).

**Synthesis of compounds (I) and (II)**

A mixture of 4-chloro­benzene­sulfono­hydrazide (0.01 mol) and 4-chloro­benzaldehyde (0.01 mol) for (I), and 4-nitro­benzaldehyde (0.01 mol) for (II), in ethanol (30 ml) and two drops of glacial acetic acid were stirred for 4 h. The reaction mixtures were cooled to room temperature and concentrated by evaporating off the excess of solvent. The solid products obtained were washed with cold water, dried and recrystallized to constant melting points from ethanol to obtain the pure compounds. The purity of the compounds was checked by TLC.

Crystals of compounds (I) and (II), suitable for X-ray diffraction analysis, were obtained by slow evaporation of their DMF solutions at room temperature.

Both compounds were characterized by measuring their IR, ^1^H and ^13^C NMR spectra.

(***E*** **)-4-Chloro-** ***N*** **′-(4-chloro­benzyl­idene)benzene­sulfono­hydrazide (I)**

Colourless rod-shaped crystals; m.p. 432–433 K; IR (cm^−1^): 3180.6 (N—H asym. stretch), 1573.9 (C=N), 1327.0 (S=O asym. stretch) and 1166.9 (S=O sym. stretch).

^1^H NMR (400 MHz, DMSO-*d_6_*): δ 7.32 (*d*, 1H, *J* = 8.4Hz, Ar-H), 7.51–7.56 (*m*, 4H, Ar-H), 7.87–7.89 (*m*, 2H, Ar-H), 7.92 (*s*, 1H), 11.50 (*s*, 1H). ^13^C NMR (100 MHz, DMSO-*d_6_*): δ 128.38, 129.42, 130.73, 132.06, 134.96, 137.47, 138.39, 139.32, 145.66.

**(** ***E*** **)-4-Chloro-** ***N*** **′-(4-nitro­benzyl­idene)benzene­sulfono­hydrazide (II)**

Yellow rod-shaped crystals; m.p. 414–415 K; IR (cm^−1^): 3093.8 (N—H asym. stretch), 1653.0 (C=N), 1392.6 (S=O asym. stretch) and 1153.4 (S=O sym. stretch).

^1^H NMR (400 MHz, DMSO-*d_6_*): δ 7.0 (*d*, 1H, *J* = 8.80, Ar-H), 7.38 (*d*, 1H, *J* = 8.52, Ar-H), 7.63 (*d*, 1H, *J* = 8.36, Ar-H), 7.64 (*s*, 1H), 7.79 (*d*, 1H, *J* = 8.56, Ar-H), 7.80 (*d*, 2H, *J* = 8.28, Ar-H), 7.90 (*s*, 1H), 11.60 (*s*, 1H). ^13^C NMR (100 MHz, DMSO-*d_6_*): δ 115.49, 124.47, 128.45, 129.63, 136.85, 137.87, 138.28, 147.97, 159.43.

## Refinement   

Crystal data, data collection and structure refinement details are summarized in Table 4 ▸. C-bound H atoms were positioned with idealized geometry and refined using a riding model: C—H = 0.93 Å with *U*_iso_(H) = 1.2*U*_eq_(C). The amino H atoms were located in difference-Fourier maps and refined with an N—H distance restraint of 0.86 (2) Å and *U*_iso_(H) = 1.2*U*_eq_(N). In (I), reflection 011 was masked by the beam stop and omitted from the refinement. In (II), atom O3 is disordered and was refined using a split model. The corresponding site-occupation factors were fixed at 0.55:0.45 and the corresponding N—O bond lengths in the disordered group were restrained to be equal. The *U* ^ij^ components of O3 and O3′ were restrained to be approximately isotropic.

## Supplementary Material

Crystal structure: contains datablock(s) I, II, global. DOI: 10.1107/S205698901801592X/su5458sup1.cif

Structure factors: contains datablock(s) I. DOI: 10.1107/S205698901801592X/su5458Isup2.hkl

Structure factors: contains datablock(s) II. DOI: 10.1107/S205698901801592X/su5458IIsup3.hkl

CCDC references: 1578709, 1578704

Additional supporting information:  crystallographic information; 3D view; checkCIF report

## Figures and Tables

**Table 1:** Comparison of selected geometrical parameters (Å, °) of compounds (I)–(V) The dihedral angle is that between the aromatic rings. The equivalent bond lengths and torsion angles are given for (II*B*).

Bond length	(I)	(II) Mol­ecule *A*	(II) Mol­ecule *B*	(III)	(IV)	(V)
C1—S1	1.763 (2)	1.754 (2)	1.760 (2)	1.752 (4)	1.751 (5)	1.761 (2)
S1—N1	1.631 (2)	1.645 (2)	1.641 (2)	1.644 (4)	1.645 (4)	1.625 (2)
N1—N2	1.388 (2)	1.397 (3)	1.390 (2)	1.394 (5)	1.407 (5)	1.393 (2)
N2—C7	1.269 (3)	1.269 (3)	1.269 (3)	1.258 (5)	1.272 (5)	1.273 (3)
C7—C8	1.463 (3)	1.465 (3)	1.462 (3)	1.473 (6)	1.461 (6)	1.458 (3)
Torsion angle						
C1—S1—N1—N2	−62.4 (2)	−46.8 (2)	56.8 (2)	−66.0 (3)	−66.0 (3)	−58.4 (2)
S1—N1—N2—C7	158.9 (2)	171.4 (2)	−165.3 (2)	166.5 (3)	165.4 (3)	157.9 (2)
N1—N2—C7—C8	175.0 (2)	−175.9 (2)	178.2 (2)	177.8 (4)	175.8 (4)	175.8 (2)
Dihedral angle	81.0 (1)	75.9 (1)	73.4 (1)	78.4 (2)	74.8 (2)	76.9 (1)

**Table 2:** Hydrogen-bond geometry (Å, °) for (I) *Cg*1 and *Cg*2 are the centroids of rings C1–C6 and C8–C13, respectively.

*D*—H⋯*A*	*D*—H	H⋯*A*	*D*⋯*A*	*D*—H⋯*A*
N1—H1*N*⋯O1^i^	0.83 (2)	2.07 (2)	2.903 (2)	178 (2)
C4—Cl1⋯*Cg*2^ii^	1.73 (1)	3.41 (1)	5.112 (2)	166 (1)
C11—Cl2⋯*Cg*1^iii^	1.74 (1)	3.65 (1)	5.372 (3)	171 (1)

Symmetry codes: (i) 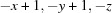; (ii) 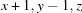; (iii) 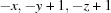.

**Table 3:** Hydrogen-bond geometry (Å, °) for (II) *Cg*3 is the centroid of the C14–C19 ring.

*D*—H⋯*A*	*D*—H	H⋯*A*	*D*⋯*A*	*D*—H⋯*A*
N1—H1*N*⋯O5^i^	0.85 (2)	2.06 (2)	2.887 (3)	163 (2)
N4—H4*N*⋯O1^ii^	0.84 (2)	2.13 (2)	2.918 (2)	157 (2)
C10—H10⋯O7^iii^	0.93	2.58	3.465 (3)	159
C16—H16⋯O4^iv^	0.93	2.58	3.259 (3)	131
C25—H25⋯O5^ii^	0.93	2.45	3.340 (3)	161
C12—H12⋯*Cg*3	0.93	2.96	3.843 (2)	160

Symmetry codes: (i) 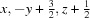; (ii) 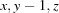; (iii) 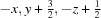; (iv) 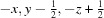.

**Table 4:** Experimental details

	(I)	(II)
Crystal data		
Chemical formula	C_13_H_10_Cl_2_N_2_O_2_S	C_13_H_10_ClN_3_O_4_S
*M*_r_	329.19	339.75
Crystal system, space group	Triclinic, *P* 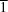	Monoclinic, *P*2_1_/*c*
Temperature (K)	293	293
*a*, *b*, *c* (Å)	5.9306 (6), 9.477 (1), 13.040 (2)	19.903 (1), 10.2517 (7), 15.064 (1)
α, β, γ (°)	98.822 (9), 96.046 (9), 92.416 (9)	90, 103.929 (7), 90
*V* (Å^3^)	718.94 (15)	2983.3 (3)
*Z*	2	8
Radiation type	Mo *K*α	Mo *K*α
μ (mm^−1^)	0.60	0.42
Crystal size (mm)	0.48 × 0.40 × 0.36	0.48 × 0.40 × 0.36

Data collection		
Diffractometer	Oxford Diffraction Xcalibur diffractometer with Sapphire CCD	Oxford Diffraction Xcalibur diffractometer with Sapphire CCD
Absorption correction	Multi-scan (*CrysAlis RED*; Oxford Diffraction, 2009 ▸)	Multi-scan (*CrysAlis RED*; Oxford Diffraction, 2009 ▸)
*T*_min_, *T*_max_	0.762, 0.814	0.825, 0.864
No. of measured, independent and observed [*I* > 2σ(*I*)] reflections	4153, 2626, 2321	19194, 5455, 4247
*R*_int_	0.016	0.025
(sin θ/λ)_max_ (Å^−1^)	0.602	0.602

Refinement		
*R*[*F* ^2^ > 2σ(*F* ^2^)], *wR*(*F* ^2^), *S*	0.038, 0.102, 1.08	0.038, 0.101, 1.02
No. of reflections	2626	5455
No. of parameters	185	414
No. of restraints	1	15
H-atom treatment	H atoms treated by a mixture of independent and constrained refinement	H atoms treated by a mixture of independent and constrained refinement
Δρ_max_, Δρ_min_ (e Å^−3^)	0.37, −0.35	0.43, −0.38

Computer programs: *CrysAlis CCD* and *CrysAlis RED* (Oxford Diffraction, 2009 ▸), *SHELXS2013* (Sheldrick, 2008 ▸), *SHELXL2014* (Sheldrick, 2015 ▸) and *PLATON* (Spek, 2009 ▸).

## supplementary crystallographic information

### (E)-4-Chloro-N'-(4-chlorobenzylidene)benzenesulfonohydrazide (I) Crystal data

**Table:**

C_13_H_10_Cl_2_N_2_O_2_S	*Z* = 2
*M**_r_* = 329.19	*F*(000) = 336
Triclinic, *P*1	*D*_x_ = 1.521 Mg m^−^^3^
*a* = 5.9306 (6) Å	Mo *K*α radiation, λ = 0.71073 Å
*b* = 9.477 (1) Å	Cell parameters from 2722 reflections
*c* = 13.040 (2) Å	θ = 2.9–27.8°
α = 98.822 (9)°	µ = 0.60 mm^−^^1^
β = 96.046 (9)°	*T* = 293 K
γ = 92.416 (9)°	Rod, colourless
*V* = 718.94 (15) Å^3^	0.48 × 0.40 × 0.36 mm

### (E)-4-Chloro-N'-(4-chlorobenzylidene)benzenesulfonohydrazide (I) Data collection

**Table:**

Oxford Diffraction Xcalibur diffractometer with Sapphire CCD	2321 reflections with *I* > 2σ(*I*)
Radiation source: Enhance (Mo) X-ray Source	*R*_int_ = 0.016
Rotation method data acquisition using ω scans.	θ_max_ = 25.4°, θ_min_ = 3.2°
Absorption correction: multi-scan (CrysAlis RED; Oxford Diffraction, 2009)	*h* = −4→7
*T*_min_ = 0.762, *T*_max_ = 0.814	*k* = −11→11
4153 measured reflections	*l* = −15→11
2626 independent reflections	

### (E)-4-Chloro-N'-(4-chlorobenzylidene)benzenesulfonohydrazide (I) Refinement

**Table:**

Refinement on *F*^2^	Hydrogen site location: mixed
Least-squares matrix: full	H atoms treated by a mixture of independent and constrained refinement
*R*[*F*^2^ > 2σ(*F*^2^)] = 0.038	*w* = 1/[σ^2^(*F*_o_^2^) + (0.0523*P*)^2^ + 0.3082*P*] where *P* = (*F*_o_^2^ + 2*F*_c_^2^)/3
*wR*(*F*^2^) = 0.102	(Δ/σ)_max_ = 0.001
*S* = 1.08	Δρ_max_ = 0.37 e Å^−^^3^
2626 reflections	Δρ_min_ = −0.35 e Å^−^^3^
185 parameters	Extinction correction: SHELXL, Fc^*^=kFc[1+0.001xFc^2^λ^3^/sin(2θ)]^-1/4^
1 restraint	Extinction coefficient: 0.025 (3)

### (E)-4-Chloro-N'-(4-chlorobenzylidene)benzenesulfonohydrazide (I) Special details

**Table:**

Geometry. All esds (except the esd in the dihedral angle between two l.s. planes) are estimated using the full covariance matrix. The cell esds are taken into account individually in the estimation of esds in distances, angles and torsion angles; correlations between esds in cell parameters are only used when they are defined by crystal symmetry. An approximate (isotropic) treatment of cell esds is used for estimating esds involving l.s. planes.

### (E)-4-Chloro-N'-(4-chlorobenzylidene)benzenesulfonohydrazide (I) Fractional atomic coordinates and isotropic or equivalent isotropic displacement parameters (Å^2^)

**Table:**

	*x*	*y*	*z*	*U*_iso_*/*U*_eq_	
C1	0.3867 (3)	0.1943 (2)	0.08046 (14)	0.0366 (4)	
C2	0.6099 (3)	0.2317 (2)	0.12163 (17)	0.0481 (5)	
H2	0.6853	0.3119	0.1052	0.058*	
C3	0.7193 (4)	0.1486 (3)	0.18737 (19)	0.0556 (6)	
H3	0.8683	0.1731	0.2166	0.067*	
C4	0.6054 (4)	0.0289 (2)	0.20921 (17)	0.0526 (6)	
C5	0.3850 (4)	−0.0103 (2)	0.16683 (18)	0.0549 (6)	
H5	0.3118	−0.0925	0.1813	0.066*	
C6	0.2746 (4)	0.0736 (2)	0.10286 (16)	0.0450 (5)	
H6	0.1248	0.0493	0.0747	0.054*	
C7	0.1757 (3)	0.5784 (2)	0.22864 (16)	0.0423 (4)	
H7	0.2965	0.6386	0.2185	0.051*	
C8	0.0609 (3)	0.6140 (2)	0.32242 (16)	0.0427 (5)	
C9	−0.1405 (4)	0.5433 (3)	0.33612 (18)	0.0542 (5)	
H9	−0.2037	0.4681	0.2855	0.065*	
C10	−0.2480 (4)	0.5832 (3)	0.4236 (2)	0.0618 (6)	
H10	−0.3837	0.5356	0.4319	0.074*	
C11	−0.1539 (4)	0.6935 (3)	0.49867 (18)	0.0575 (6)	
C12	0.0449 (4)	0.7658 (3)	0.48740 (19)	0.0630 (6)	
H12	0.1068	0.8410	0.5383	0.076*	
C13	0.1518 (4)	0.7252 (3)	0.39922 (18)	0.0548 (6)	
H13	0.2872	0.7734	0.3913	0.066*	
N1	0.2346 (3)	0.45985 (18)	0.07324 (14)	0.0426 (4)	
H1N	0.345 (3)	0.517 (2)	0.0743 (19)	0.051*	
N2	0.1173 (3)	0.46871 (18)	0.16049 (13)	0.0414 (4)	
O1	0.3856 (3)	0.33553 (16)	−0.07638 (11)	0.0487 (4)	
O2	0.0199 (2)	0.24297 (16)	−0.03067 (12)	0.0492 (4)	
Cl1	0.74291 (16)	−0.07477 (8)	0.29214 (6)	0.0886 (3)	
Cl2	−0.29271 (14)	0.74500 (11)	0.60842 (6)	0.0909 (3)	
S1	0.24404 (8)	0.30435 (5)	0.00069 (4)	0.03746 (17)	

### (E)-4-Chloro-N'-(4-chlorobenzylidene)benzenesulfonohydrazide (I) Atomic displacement parameters (Å^2^)

**Table:**

	*U*^11^	*U*^22^	*U*^33^	*U*^12^	*U*^13^	*U*^23^
C1	0.0347 (9)	0.0351 (9)	0.0370 (9)	−0.0002 (7)	0.0035 (7)	−0.0024 (8)
C2	0.0372 (10)	0.0498 (12)	0.0547 (12)	−0.0056 (9)	0.0048 (9)	0.0032 (10)
C3	0.0394 (11)	0.0632 (15)	0.0580 (13)	0.0069 (10)	−0.0054 (10)	−0.0029 (11)
C4	0.0661 (14)	0.0415 (11)	0.0443 (11)	0.0170 (10)	−0.0079 (10)	−0.0060 (9)
C5	0.0682 (15)	0.0346 (11)	0.0581 (13)	−0.0040 (10)	−0.0038 (11)	0.0044 (9)
C6	0.0433 (11)	0.0377 (10)	0.0499 (11)	−0.0066 (8)	−0.0053 (9)	0.0033 (9)
C7	0.0429 (11)	0.0383 (10)	0.0459 (11)	0.0015 (8)	0.0056 (9)	0.0078 (9)
C8	0.0460 (11)	0.0406 (10)	0.0420 (11)	0.0083 (8)	0.0036 (9)	0.0068 (8)
C9	0.0571 (13)	0.0519 (13)	0.0501 (12)	−0.0022 (10)	0.0100 (10)	−0.0040 (10)
C10	0.0570 (14)	0.0708 (16)	0.0574 (14)	0.0003 (12)	0.0175 (11)	0.0030 (12)
C11	0.0581 (14)	0.0723 (16)	0.0416 (11)	0.0186 (12)	0.0071 (10)	0.0023 (11)
C12	0.0666 (16)	0.0666 (16)	0.0473 (13)	0.0053 (12)	−0.0027 (11)	−0.0117 (11)
C13	0.0522 (13)	0.0573 (13)	0.0512 (12)	0.0001 (10)	0.0031 (10)	−0.0004 (10)
N1	0.0459 (9)	0.0357 (9)	0.0461 (9)	−0.0045 (7)	0.0133 (8)	0.0032 (7)
N2	0.0426 (9)	0.0396 (9)	0.0438 (9)	0.0039 (7)	0.0110 (7)	0.0074 (7)
O1	0.0569 (9)	0.0475 (8)	0.0396 (7)	−0.0107 (7)	0.0117 (6)	0.0005 (6)
O2	0.0413 (8)	0.0474 (8)	0.0559 (9)	−0.0080 (6)	−0.0055 (6)	0.0093 (7)
Cl1	0.1200 (7)	0.0595 (4)	0.0754 (5)	0.0290 (4)	−0.0372 (4)	0.0025 (3)
Cl2	0.0868 (5)	0.1270 (7)	0.0546 (4)	0.0193 (5)	0.0230 (4)	−0.0133 (4)
S1	0.0382 (3)	0.0352 (3)	0.0372 (3)	−0.00503 (18)	0.00356 (19)	0.00245 (19)

### (E)-4-Chloro-N'-(4-chlorobenzylidene)benzenesulfonohydrazide (I) Geometric parameters (Å, º)

**Table:**

C1—C6	1.384 (3)	C8—C9	1.386 (3)
C1—C2	1.385 (3)	C9—C10	1.376 (3)
C1—S1	1.763 (2)	C9—H9	0.9300
C2—C3	1.382 (3)	C10—C11	1.373 (4)
C2—H2	0.9300	C10—H10	0.9300
C3—C4	1.377 (4)	C11—C12	1.371 (4)
C3—H3	0.9300	C11—Cl2	1.741 (2)
C4—C5	1.377 (3)	C12—C13	1.382 (3)
C4—Cl1	1.734 (2)	C12—H12	0.9300
C5—C6	1.374 (3)	C13—H13	0.9300
C5—H5	0.9300	N1—N2	1.388 (2)
C6—H6	0.9300	N1—S1	1.6311 (17)
C7—N2	1.269 (3)	N1—H1N	0.830 (16)
C7—C8	1.463 (3)	O1—S1	1.4316 (14)
C7—H7	0.9300	O2—S1	1.4212 (15)
C8—C13	1.385 (3)		
			
C6—C1—C2	120.77 (19)	C10—C9—H9	119.6
C6—C1—S1	120.05 (15)	C8—C9—H9	119.6
C2—C1—S1	119.18 (15)	C11—C10—C9	119.6 (2)
C3—C2—C1	119.3 (2)	C11—C10—H10	120.2
C3—C2—H2	120.4	C9—C10—H10	120.2
C1—C2—H2	120.4	C12—C11—C10	121.0 (2)
C4—C3—C2	119.3 (2)	C12—C11—Cl2	119.5 (2)
C4—C3—H3	120.3	C10—C11—Cl2	119.4 (2)
C2—C3—H3	120.3	C11—C12—C13	119.0 (2)
C5—C4—C3	121.6 (2)	C11—C12—H12	120.5
C5—C4—Cl1	119.19 (19)	C13—C12—H12	120.5
C3—C4—Cl1	119.22 (18)	C12—C13—C8	121.2 (2)
C6—C5—C4	119.2 (2)	C12—C13—H13	119.4
C6—C5—H5	120.4	C8—C13—H13	119.4
C4—C5—H5	120.4	N2—N1—S1	118.76 (13)
C5—C6—C1	119.84 (19)	N2—N1—H1N	118.8 (17)
C5—C6—H6	120.1	S1—N1—H1N	115.6 (17)
C1—C6—H6	120.1	C7—N2—N1	114.02 (16)
N2—C7—C8	122.74 (19)	O2—S1—O1	119.93 (9)
N2—C7—H7	118.6	O2—S1—N1	109.69 (9)
C8—C7—H7	118.6	O1—S1—N1	102.85 (9)
C13—C8—C9	118.4 (2)	O2—S1—C1	107.94 (9)
C13—C8—C7	119.19 (19)	O1—S1—C1	109.14 (9)
C9—C8—C7	122.40 (19)	N1—S1—C1	106.52 (9)
C10—C9—C8	120.8 (2)		
			
C6—C1—C2—C3	−1.2 (3)	C10—C11—C12—C13	−0.6 (4)
S1—C1—C2—C3	177.57 (16)	Cl2—C11—C12—C13	−179.08 (19)
C1—C2—C3—C4	1.1 (3)	C11—C12—C13—C8	0.4 (4)
C2—C3—C4—C5	0.2 (3)	C9—C8—C13—C12	−0.3 (4)
C2—C3—C4—Cl1	−179.75 (17)	C7—C8—C13—C12	177.7 (2)
C3—C4—C5—C6	−1.3 (4)	C8—C7—N2—N1	175.01 (17)
Cl1—C4—C5—C6	178.64 (17)	S1—N1—N2—C7	158.96 (15)
C4—C5—C6—C1	1.1 (3)	N2—N1—S1—O2	54.13 (17)
C2—C1—C6—C5	0.1 (3)	N2—N1—S1—O1	−177.17 (15)
S1—C1—C6—C5	−178.67 (17)	N2—N1—S1—C1	−62.44 (17)
N2—C7—C8—C13	172.1 (2)	C6—C1—S1—O2	−0.97 (19)
N2—C7—C8—C9	−10.0 (3)	C2—C1—S1—O2	−179.78 (15)
C13—C8—C9—C10	0.3 (4)	C6—C1—S1—O1	−132.84 (16)
C7—C8—C9—C10	−177.6 (2)	C2—C1—S1—O1	48.35 (18)
C8—C9—C10—C11	−0.5 (4)	C6—C1—S1—N1	116.77 (17)
C9—C10—C11—C12	0.7 (4)	C2—C1—S1—N1	−62.04 (18)
C9—C10—C11—Cl2	179.13 (19)		

### (E)-4-Chloro-N'-(4-chlorobenzylidene)benzenesulfonohydrazide (I) Hydrogen-bond geometry (Å, º)

Cg1 and Cg2 are the centroids of rings C1–C6 and C8–C13, respectively.

**Table:**

*D*—H···*A*	*D*—H	H···*A*	*D*···*A*	*D*—H···*A*
N1—H1*N*···O1^i^	0.83 (2)	2.07 (2)	2.903 (2)	178 (2)
C4—Cl1···*Cg*2^ii^	1.73 (1)	3.41 (1)	5.112 (2)	166 (1)
C11—Cl2···*Cg*1^iii^	1.74 (1)	3.65 (1)	5.372 (3)	171 (1)

Symmetry codes: (i) −*x*+1, −*y*+1, −*z*; (ii) *x*+1, *y*−1, *z*; (iii) −*x*, −*y*+1, −*z*+1.

### (E)-4-chloro-N'-(4-Nitrobenzylidene)benzenesulfonohydrazide (II) Crystal data

**Table:**

C_13_H_10_ClN_3_O_4_S	*F*(000) = 1392
*M**_r_* = 339.75	*D*_x_ = 1.513 Mg m^−^^3^
Monoclinic, *P*2_1_/*c*	Mo *K*α radiation, λ = 0.71073 Å
*a* = 19.903 (1) Å	Cell parameters from 7054 reflections
*b* = 10.2517 (7) Å	θ = 2.8–27.8°
*c* = 15.064 (1) Å	µ = 0.42 mm^−^^1^
β = 103.929 (7)°	*T* = 293 K
*V* = 2983.3 (3) Å^3^	Rod, yellow
*Z* = 8	0.48 × 0.40 × 0.36 mm

### (E)-4-chloro-N'-(4-Nitrobenzylidene)benzenesulfonohydrazide (II) Data collection

**Table:**

Oxford Diffraction Xcalibur diffractometer with Sapphire CCD	4247 reflections with *I* > 2σ(*I*)
Radiation source: Enhance (Mo) X-ray Source	*R*_int_ = 0.025
Rotation method data acquisition using ω scans.	θ_max_ = 25.4°, θ_min_ = 2.8°
Absorption correction: multi-scan (CrysAlis RED; Oxford Diffraction, 2009)	*h* = −23→23
*T*_min_ = 0.825, *T*_max_ = 0.864	*k* = −12→12
19194 measured reflections	*l* = −18→18
5455 independent reflections	

### (E)-4-chloro-N'-(4-Nitrobenzylidene)benzenesulfonohydrazide (II) Refinement

**Table:**

Refinement on *F*^2^	Hydrogen site location: mixed
Least-squares matrix: full	H atoms treated by a mixture of independent and constrained refinement
*R*[*F*^2^ > 2σ(*F*^2^)] = 0.038	*w* = 1/[σ^2^(*F*_o_^2^) + (0.0435*P*)^2^ + 1.8451*P*] where *P* = (*F*_o_^2^ + 2*F*_c_^2^)/3
*wR*(*F*^2^) = 0.101	(Δ/σ)_max_ = 0.001
*S* = 1.02	Δρ_max_ = 0.43 e Å^−^^3^
5455 reflections	Δρ_min_ = −0.38 e Å^−^^3^
414 parameters	Extinction correction: SHELXL, Fc^*^=kFc[1+0.001xFc^2^λ^3^/sin(2θ)]^-1/4^
15 restraints	Extinction coefficient: 0.0034 (3)

### (E)-4-chloro-N'-(4-Nitrobenzylidene)benzenesulfonohydrazide (II) Special details

**Table:**

Geometry. All esds (except the esd in the dihedral angle between two l.s. planes) are estimated using the full covariance matrix. The cell esds are taken into account individually in the estimation of esds in distances, angles and torsion angles; correlations between esds in cell parameters are only used when they are defined by crystal symmetry. An approximate (isotropic) treatment of cell esds is used for estimating esds involving l.s. planes.

### (E)-4-chloro-N'-(4-Nitrobenzylidene)benzenesulfonohydrazide (II) Fractional atomic coordinates and isotropic or equivalent isotropic displacement parameters (Å^2^)

**Table:**

	*x*	*y*	*z*	*U*_iso_*/*U*_eq_	Occ. (<1)
Cl1	0.44715 (4)	0.44299 (7)	0.63321 (5)	0.0692 (2)	
S1	0.41281 (3)	1.03308 (5)	0.53686 (4)	0.03843 (15)	
O1	0.42255 (8)	1.04883 (15)	0.44626 (10)	0.0447 (4)	
O2	0.44990 (8)	1.11288 (16)	0.60973 (11)	0.0529 (4)	
O3	0.0285 (6)	0.5036 (8)	0.3381 (6)	0.081 (3)	0.55
O3'	0.0324 (8)	0.5149 (10)	0.3044 (7)	0.075 (3)	0.45
O4	−0.04648 (9)	0.6576 (2)	0.30599 (13)	0.0681 (5)	
N1	0.33087 (10)	1.06413 (19)	0.53180 (14)	0.0449 (5)	
H1N	0.3271 (13)	1.086 (2)	0.5849 (12)	0.054*	
N2	0.28443 (9)	0.97344 (18)	0.48183 (12)	0.0426 (4)	
N3	0.01310 (11)	0.6206 (2)	0.32944 (15)	0.0562 (5)	
C1	0.42530 (10)	0.8671 (2)	0.56436 (14)	0.0367 (5)	
C2	0.44115 (12)	0.8290 (2)	0.65534 (15)	0.0465 (6)	
H2	0.4461	0.8912	0.7014	0.056*	
C3	0.44953 (12)	0.6982 (3)	0.67705 (16)	0.0519 (6)	
H3	0.4610	0.6714	0.7378	0.062*	
C4	0.44062 (11)	0.6085 (2)	0.60760 (17)	0.0457 (6)	
C5	0.42596 (13)	0.6447 (2)	0.51720 (17)	0.0534 (6)	
H5	0.4209	0.5821	0.4714	0.064*	
C6	0.41891 (13)	0.7760 (2)	0.49545 (15)	0.0484 (6)	
H6	0.4099	0.8027	0.4347	0.058*	
C7	0.22211 (11)	0.9867 (2)	0.48701 (16)	0.0438 (5)	
H7	0.2100	1.0569	0.5192	0.053*	
C8	0.16896 (11)	0.8929 (2)	0.44293 (15)	0.0406 (5)	
C9	0.09969 (12)	0.9292 (2)	0.42131 (17)	0.0499 (6)	
H9	0.0877	1.0142	0.4327	0.060*	
C10	0.04837 (12)	0.8412 (2)	0.38318 (17)	0.0497 (6)	
H10	0.0021	0.8661	0.3679	0.060*	
C11	0.06736 (11)	0.7159 (2)	0.36837 (16)	0.0443 (5)	
C12	0.13570 (12)	0.6763 (2)	0.38937 (18)	0.0508 (6)	
H12	0.1472	0.5907	0.3787	0.061*	
C13	0.18635 (12)	0.7649 (2)	0.42624 (17)	0.0476 (6)	
H13	0.2326	0.7395	0.4402	0.057*	
Cl2	0.11071 (4)	0.30845 (9)	0.53034 (6)	0.0814 (3)	
S2	0.32436 (3)	0.34333 (5)	0.28991 (4)	0.04203 (16)	
O5	0.28924 (9)	0.38026 (17)	0.19868 (11)	0.0542 (4)	
O6	0.38459 (9)	0.41231 (17)	0.33703 (13)	0.0610 (5)	
O7	0.10708 (11)	−0.49600 (19)	0.13632 (16)	0.0778 (6)	
O8	0.03109 (10)	−0.3435 (2)	0.12357 (18)	0.0896 (7)	
N4	0.34961 (10)	0.19182 (18)	0.28409 (13)	0.0413 (4)	
H4N	0.3793 (11)	0.167 (2)	0.3306 (13)	0.050*	
N5	0.29454 (9)	0.10878 (17)	0.24828 (12)	0.0386 (4)	
N6	0.09076 (11)	−0.3814 (2)	0.14089 (16)	0.0582 (6)	
C14	0.26262 (11)	0.3420 (2)	0.35620 (15)	0.0388 (5)	
C15	0.19424 (12)	0.3099 (2)	0.31583 (15)	0.0432 (5)	
H15	0.1803	0.2948	0.2532	0.052*	
C16	0.14730 (12)	0.3006 (2)	0.36964 (17)	0.0479 (6)	
H16	0.1014	0.2790	0.3437	0.058*	
C17	0.16929 (13)	0.3237 (2)	0.46229 (17)	0.0483 (6)	
C18	0.23655 (13)	0.3573 (3)	0.50252 (16)	0.0534 (6)	
H18	0.2500	0.3742	0.5650	0.064*	
C19	0.28388 (12)	0.3655 (2)	0.44925 (16)	0.0489 (6)	
H19	0.3298	0.3868	0.4757	0.059*	
C20	0.30626 (12)	−0.0126 (2)	0.25909 (15)	0.0413 (5)	
H20	0.3503	−0.0419	0.2881	0.050*	
C21	0.25043 (11)	−0.1061 (2)	0.22580 (15)	0.0395 (5)	
C22	0.18148 (12)	−0.0665 (2)	0.20002 (18)	0.0496 (6)	
H22	0.1707	0.0215	0.2026	0.060*	
C23	0.12929 (12)	−0.1552 (2)	0.17098 (18)	0.0511 (6)	
H23	0.0834	−0.1283	0.1534	0.061*	
C24	0.14638 (12)	−0.2851 (2)	0.16848 (16)	0.0436 (5)	
C25	0.21363 (12)	−0.3280 (2)	0.19346 (17)	0.0498 (6)	
H25	0.2238	−0.4163	0.1907	0.060*	
C26	0.26565 (12)	−0.2384 (2)	0.22262 (17)	0.0482 (6)	
H26	0.3114	−0.2663	0.2404	0.058*	

### (E)-4-chloro-N'-(4-Nitrobenzylidene)benzenesulfonohydrazide (II) Atomic displacement parameters (Å^2^)

**Table:**

	*U*^11^	*U*^22^	*U*^33^	*U*^12^	*U*^13^	*U*^23^
Cl1	0.0651 (4)	0.0455 (4)	0.0853 (5)	−0.0022 (3)	−0.0048 (4)	0.0214 (3)
S1	0.0359 (3)	0.0367 (3)	0.0404 (3)	−0.0048 (2)	0.0046 (2)	−0.0003 (2)
O1	0.0414 (8)	0.0469 (9)	0.0454 (9)	−0.0022 (7)	0.0096 (7)	0.0086 (7)
O2	0.0544 (10)	0.0450 (10)	0.0524 (10)	−0.0110 (8)	−0.0006 (8)	−0.0087 (8)
O3	0.058 (3)	0.050 (3)	0.129 (7)	−0.007 (2)	0.010 (5)	−0.002 (4)
O3'	0.069 (4)	0.062 (4)	0.087 (5)	−0.005 (4)	0.005 (4)	−0.018 (4)
O4	0.0382 (10)	0.0845 (15)	0.0791 (13)	−0.0053 (9)	0.0093 (9)	−0.0026 (11)
N1	0.0406 (10)	0.0446 (11)	0.0488 (11)	−0.0001 (9)	0.0094 (9)	−0.0083 (9)
N2	0.0380 (10)	0.0443 (11)	0.0438 (10)	−0.0011 (9)	0.0065 (8)	−0.0028 (9)
N3	0.0421 (12)	0.0600 (15)	0.0649 (14)	−0.0050 (11)	0.0102 (10)	−0.0023 (12)
C1	0.0310 (10)	0.0404 (12)	0.0371 (11)	−0.0048 (9)	0.0051 (8)	0.0017 (9)
C2	0.0483 (13)	0.0506 (14)	0.0360 (12)	−0.0052 (11)	0.0014 (10)	−0.0009 (10)
C3	0.0507 (14)	0.0594 (16)	0.0393 (13)	−0.0033 (12)	−0.0015 (11)	0.0136 (12)
C4	0.0367 (12)	0.0401 (13)	0.0557 (14)	−0.0023 (10)	0.0021 (10)	0.0126 (11)
C5	0.0664 (16)	0.0405 (13)	0.0511 (14)	−0.0042 (12)	0.0097 (12)	−0.0031 (11)
C6	0.0650 (15)	0.0447 (14)	0.0337 (12)	−0.0035 (12)	0.0085 (11)	0.0028 (10)
C7	0.0400 (12)	0.0392 (12)	0.0527 (14)	0.0061 (10)	0.0119 (10)	0.0010 (10)
C8	0.0355 (11)	0.0437 (13)	0.0434 (12)	0.0033 (10)	0.0113 (9)	0.0039 (10)
C9	0.0397 (12)	0.0435 (13)	0.0667 (16)	0.0103 (11)	0.0131 (11)	−0.0002 (12)
C10	0.0324 (11)	0.0535 (15)	0.0614 (15)	0.0076 (11)	0.0081 (11)	0.0050 (12)
C11	0.0383 (12)	0.0475 (14)	0.0472 (13)	−0.0010 (10)	0.0105 (10)	0.0034 (11)
C12	0.0419 (13)	0.0418 (13)	0.0677 (16)	0.0059 (11)	0.0113 (11)	−0.0033 (12)
C13	0.0327 (11)	0.0481 (14)	0.0616 (15)	0.0079 (10)	0.0106 (10)	0.0033 (12)
Cl2	0.0824 (5)	0.0998 (6)	0.0759 (5)	−0.0171 (5)	0.0459 (4)	−0.0163 (4)
S2	0.0445 (3)	0.0341 (3)	0.0487 (3)	−0.0011 (2)	0.0137 (2)	0.0007 (2)
O5	0.0642 (11)	0.0510 (10)	0.0514 (10)	0.0132 (9)	0.0218 (8)	0.0169 (8)
O6	0.0524 (10)	0.0512 (10)	0.0824 (13)	−0.0141 (8)	0.0219 (9)	−0.0147 (9)
O7	0.0715 (13)	0.0450 (11)	0.1165 (18)	−0.0122 (10)	0.0217 (12)	−0.0072 (11)
O8	0.0452 (11)	0.0735 (15)	0.143 (2)	−0.0107 (10)	0.0096 (12)	−0.0044 (14)
N4	0.0401 (10)	0.0353 (10)	0.0446 (11)	0.0022 (8)	0.0027 (8)	−0.0006 (8)
N5	0.0395 (10)	0.0349 (10)	0.0397 (10)	−0.0002 (8)	0.0064 (8)	0.0001 (8)
N6	0.0521 (13)	0.0532 (14)	0.0700 (15)	−0.0095 (11)	0.0160 (11)	−0.0011 (11)
C14	0.0439 (12)	0.0315 (11)	0.0407 (12)	0.0031 (9)	0.0094 (9)	−0.0009 (9)
C15	0.0451 (13)	0.0432 (13)	0.0388 (12)	0.0024 (10)	0.0050 (10)	−0.0056 (10)
C16	0.0425 (13)	0.0468 (14)	0.0533 (14)	−0.0016 (11)	0.0093 (11)	−0.0062 (11)
C17	0.0542 (14)	0.0443 (13)	0.0511 (14)	−0.0032 (11)	0.0216 (11)	−0.0061 (11)
C18	0.0642 (16)	0.0565 (16)	0.0388 (13)	−0.0021 (13)	0.0110 (11)	−0.0084 (11)
C19	0.0462 (13)	0.0501 (14)	0.0474 (14)	−0.0060 (11)	0.0051 (11)	−0.0078 (11)
C20	0.0411 (12)	0.0405 (13)	0.0419 (12)	0.0052 (10)	0.0092 (10)	0.0001 (10)
C21	0.0423 (12)	0.0346 (12)	0.0413 (12)	0.0043 (10)	0.0094 (10)	0.0013 (10)
C22	0.0452 (13)	0.0356 (12)	0.0669 (16)	0.0070 (10)	0.0112 (12)	0.0032 (11)
C23	0.0389 (12)	0.0443 (14)	0.0683 (16)	0.0054 (11)	0.0089 (11)	0.0055 (12)
C24	0.0441 (13)	0.0399 (13)	0.0486 (13)	−0.0041 (10)	0.0149 (10)	−0.0008 (10)
C25	0.0517 (14)	0.0334 (12)	0.0660 (16)	0.0041 (11)	0.0173 (12)	−0.0051 (11)
C26	0.0404 (12)	0.0403 (13)	0.0631 (15)	0.0058 (10)	0.0107 (11)	−0.0031 (11)

### (E)-4-chloro-N'-(4-Nitrobenzylidene)benzenesulfonohydrazide (II) Geometric parameters (Å, º)

**Table:**

Cl1—C4	1.738 (2)	Cl2—C17	1.735 (2)
S1—O2	1.4245 (16)	S2—O6	1.4252 (17)
S1—O1	1.4331 (16)	S2—O5	1.4346 (17)
S1—N1	1.6452 (19)	S2—N4	1.6414 (19)
S1—C1	1.754 (2)	S2—C14	1.760 (2)
O3—N3	1.237 (8)	O7—N6	1.226 (3)
O3'—N3	1.239 (10)	O8—N6	1.217 (3)
O4—N3	1.213 (3)	N4—N5	1.390 (2)
N1—N2	1.397 (3)	N4—H4N	0.839 (16)
N1—H1N	0.849 (16)	N5—C20	1.269 (3)
N2—C7	1.269 (3)	N6—C24	1.467 (3)
N3—C11	1.469 (3)	C14—C19	1.384 (3)
C1—C6	1.379 (3)	C14—C15	1.390 (3)
C1—C2	1.386 (3)	C15—C16	1.380 (3)
C2—C3	1.381 (3)	C15—H15	0.9300
C2—H2	0.9300	C16—C17	1.379 (3)
C3—C4	1.372 (4)	C16—H16	0.9300
C3—H3	0.9300	C17—C18	1.374 (3)
C4—C5	1.373 (3)	C18—C19	1.379 (3)
C5—C6	1.385 (3)	C18—H18	0.9300
C5—H5	0.9300	C19—H19	0.9300
C6—H6	0.9300	C20—C21	1.462 (3)
C7—C8	1.465 (3)	C20—H20	0.9300
C7—H7	0.9300	C21—C26	1.393 (3)
C8—C9	1.389 (3)	C21—C22	1.394 (3)
C8—C13	1.396 (3)	C22—C23	1.370 (3)
C9—C10	1.380 (3)	C22—H22	0.9300
C9—H9	0.9300	C23—C24	1.377 (3)
C10—C11	1.372 (3)	C23—H23	0.9300
C10—H10	0.9300	C24—C25	1.373 (3)
C11—C12	1.381 (3)	C25—C26	1.375 (3)
C12—C13	1.371 (3)	C25—H25	0.9300
C12—H12	0.9300	C26—H26	0.9300
C13—H13	0.9300		
			
O2—S1—O1	120.35 (10)	C8—C13—H13	119.8
O2—S1—N1	104.40 (10)	O6—S2—O5	120.24 (11)
O1—S1—N1	107.10 (10)	O6—S2—N4	105.33 (10)
O2—S1—C1	110.93 (10)	O5—S2—N4	106.46 (10)
O1—S1—C1	106.87 (10)	O6—S2—C14	109.87 (10)
N1—S1—C1	106.33 (10)	O5—S2—C14	107.46 (10)
N2—N1—S1	114.59 (15)	N4—S2—C14	106.70 (10)
N2—N1—H1N	118.9 (18)	N5—N4—S2	112.16 (14)
S1—N1—H1N	108.5 (17)	N5—N4—H4N	118.0 (17)
C7—N2—N1	115.17 (19)	S2—N4—H4N	113.8 (17)
O4—N3—O3	122.2 (6)	C20—N5—N4	116.56 (18)
O4—N3—O3'	122.5 (8)	O8—N6—O7	123.5 (2)
O4—N3—C11	118.8 (2)	O8—N6—C24	118.5 (2)
O3—N3—C11	117.5 (6)	O7—N6—C24	118.0 (2)
O3'—N3—C11	116.8 (8)	C19—C14—C15	120.8 (2)
C6—C1—C2	120.7 (2)	C19—C14—S2	119.21 (17)
C6—C1—S1	119.78 (17)	C15—C14—S2	119.83 (17)
C2—C1—S1	119.49 (17)	C16—C15—C14	119.3 (2)
C3—C2—C1	119.6 (2)	C16—C15—H15	120.3
C3—C2—H2	120.2	C14—C15—H15	120.3
C1—C2—H2	120.2	C17—C16—C15	119.2 (2)
C4—C3—C2	119.0 (2)	C17—C16—H16	120.4
C4—C3—H3	120.5	C15—C16—H16	120.4
C2—C3—H3	120.5	C18—C17—C16	121.9 (2)
C3—C4—C5	122.2 (2)	C18—C17—Cl2	118.95 (19)
C3—C4—Cl1	119.75 (19)	C16—C17—Cl2	119.19 (19)
C5—C4—Cl1	118.1 (2)	C17—C18—C19	119.2 (2)
C4—C5—C6	118.8 (2)	C17—C18—H18	120.4
C4—C5—H5	120.6	C19—C18—H18	120.4
C6—C5—H5	120.6	C18—C19—C14	119.6 (2)
C1—C6—C5	119.6 (2)	C18—C19—H19	120.2
C1—C6—H6	120.2	C14—C19—H19	120.2
C5—C6—H6	120.2	N5—C20—C21	119.9 (2)
N2—C7—C8	120.8 (2)	N5—C20—H20	120.1
N2—C7—H7	119.6	C21—C20—H20	120.1
C8—C7—H7	119.6	C26—C21—C22	118.8 (2)
C9—C8—C13	119.0 (2)	C26—C21—C20	119.8 (2)
C9—C8—C7	119.9 (2)	C22—C21—C20	121.3 (2)
C13—C8—C7	121.0 (2)	C23—C22—C21	121.0 (2)
C10—C9—C8	121.0 (2)	C23—C22—H22	119.5
C10—C9—H9	119.5	C21—C22—H22	119.5
C8—C9—H9	119.5	C22—C23—C24	118.5 (2)
C11—C10—C9	118.3 (2)	C22—C23—H23	120.8
C11—C10—H10	120.8	C24—C23—H23	120.8
C9—C10—H10	120.8	C25—C24—C23	122.3 (2)
C10—C11—C12	122.2 (2)	C25—C24—N6	118.8 (2)
C10—C11—N3	118.8 (2)	C23—C24—N6	118.9 (2)
C12—C11—N3	119.0 (2)	C24—C25—C26	118.9 (2)
C13—C12—C11	119.0 (2)	C24—C25—H25	120.6
C13—C12—H12	120.5	C26—C25—H25	120.6
C11—C12—H12	120.5	C25—C26—C21	120.6 (2)
C12—C13—C8	120.4 (2)	C25—C26—H26	119.7
C12—C13—H13	119.8	C21—C26—H26	119.7
			
O2—S1—N1—N2	−164.14 (15)	C7—C8—C13—C12	176.5 (2)
O1—S1—N1—N2	67.20 (18)	O6—S2—N4—N5	173.52 (15)
C1—S1—N1—N2	−46.79 (18)	O5—S2—N4—N5	−57.76 (17)
S1—N1—N2—C7	171.43 (17)	C14—S2—N4—N5	56.77 (17)
O2—S1—C1—C6	−153.93 (18)	S2—N4—N5—C20	−165.33 (16)
O1—S1—C1—C6	−21.0 (2)	O6—S2—C14—C19	−19.8 (2)
N1—S1—C1—C6	93.2 (2)	O5—S2—C14—C19	−152.28 (18)
O2—S1—C1—C2	26.4 (2)	N4—S2—C14—C19	93.9 (2)
O1—S1—C1—C2	159.36 (17)	O6—S2—C14—C15	163.92 (18)
N1—S1—C1—C2	−86.50 (19)	O5—S2—C14—C15	31.5 (2)
C6—C1—C2—C3	−1.0 (3)	N4—S2—C14—C15	−82.38 (19)
S1—C1—C2—C3	178.61 (18)	C19—C14—C15—C16	−0.5 (3)
C1—C2—C3—C4	−1.2 (4)	S2—C14—C15—C16	175.69 (17)
C2—C3—C4—C5	2.3 (4)	C14—C15—C16—C17	0.2 (3)
C2—C3—C4—Cl1	−177.17 (18)	C15—C16—C17—C18	0.8 (4)
C3—C4—C5—C6	−1.0 (4)	C15—C16—C17—Cl2	−178.57 (18)
Cl1—C4—C5—C6	178.45 (19)	C16—C17—C18—C19	−1.3 (4)
C2—C1—C6—C5	2.3 (4)	Cl2—C17—C18—C19	178.0 (2)
S1—C1—C6—C5	−177.32 (19)	C17—C18—C19—C14	1.0 (4)
C4—C5—C6—C1	−1.3 (4)	C15—C14—C19—C18	−0.1 (4)
N1—N2—C7—C8	−175.87 (19)	S2—C14—C19—C18	−176.29 (19)
N2—C7—C8—C9	−157.7 (2)	N4—N5—C20—C21	178.17 (18)
N2—C7—C8—C13	25.5 (3)	N5—C20—C21—C26	168.5 (2)
C13—C8—C9—C10	−0.5 (4)	N5—C20—C21—C22	−13.9 (3)
C7—C8—C9—C10	−177.3 (2)	C26—C21—C22—C23	−0.7 (4)
C8—C9—C10—C11	1.0 (4)	C20—C21—C22—C23	−178.4 (2)
C9—C10—C11—C12	−0.7 (4)	C21—C22—C23—C24	0.5 (4)
C9—C10—C11—N3	178.8 (2)	C22—C23—C24—C25	−0.4 (4)
O4—N3—C11—C10	4.8 (3)	C22—C23—C24—N6	177.7 (2)
O3—N3—C11—C10	−161.9 (5)	O8—N6—C24—C25	176.1 (2)
O3'—N3—C11—C10	169.5 (6)	O7—N6—C24—C25	−4.1 (4)
O4—N3—C11—C12	−175.6 (2)	O8—N6—C24—C23	−2.1 (4)
O3—N3—C11—C12	17.7 (6)	O7—N6—C24—C23	177.8 (2)
O3'—N3—C11—C12	−10.9 (6)	C23—C24—C25—C26	0.5 (4)
C10—C11—C12—C13	0.0 (4)	N6—C24—C25—C26	−177.6 (2)
N3—C11—C12—C13	−179.6 (2)	C24—C25—C26—C21	−0.7 (4)
C11—C12—C13—C8	0.5 (4)	C22—C21—C26—C25	0.8 (4)
C9—C8—C13—C12	−0.2 (4)	C20—C21—C26—C25	178.5 (2)

### (E)-4-chloro-N'-(4-Nitrobenzylidene)benzenesulfonohydrazide (II) Hydrogen-bond geometry (Å, º)

Cg3 is the centroid of the C14–C19 ring.

**Table:**

*D*—H···*A*	*D*—H	H···*A*	*D*···*A*	*D*—H···*A*
N1—H1*N*···O5^i^	0.85 (2)	2.06 (2)	2.887 (3)	163 (2)
N4—H4*N*···O1^ii^	0.84 (2)	2.13 (2)	2.918 (2)	157 (2)
C10—H10···O7^iii^	0.93	2.58	3.465 (3)	159
C16—H16···O4^iv^	0.93	2.58	3.259 (3)	131
C25—H25···O5^ii^	0.93	2.45	3.340 (3)	161
C12—H12···*Cg*3	0.93	2.96	3.843 (2)	160

Symmetry codes: (i) *x*, −*y*+3/2, *z*+1/2; (ii) *x*, *y*−1, *z*; (iii) −*x*, *y*+3/2, −*z*+1/2; (iv) −*x*, *y*−1/2, −*z*+1/2.
